# Treatment of [99mTc]Tc‐hydroxy‐diphosphonate ([99mTc]Tc‐HDP) extravasation using hyaluronidase

**DOI:** 10.1002/prp2.1232

**Published:** 2024-07-25

**Authors:** Kevin R. Doornhof, Quido de Lussanet de la Sablonière, Stijn L. W. Koolen, Mark W. Konijnenberg

**Affiliations:** ^1^ Department of Radiology and Nuclear Medicine, Erasmus Medical Center University Medical Center Rotterdam Rotterdam The Netherlands

**Keywords:** bone scintigraphy, extravasation, radiology [99mTc]Tc‐HDP

## Abstract

Extravasation of 99mTc‐labeled radiopharmaceuticals is generally considered to require no specific intervention. In the presented case, the use of hyaluronidase could have minimized the adverse effects resulting from such an extravasation. Currently, no guidelines exist regarding the use of hyaluronidase after extravasation of [99mTc]Tc‐HDP. Considering the low risk of administering hyaluronidase, it should be considered to limit the risk of injury after extravasation of [99mTc]Tc‐HDP.

## Graphical abstract

The spreading of [99mTC]Tc‐HDP after extravasation can be facilitated through administration of hyaluronidase. This can significantly reduce the tissue absorbed dose, potentially reducing the severity of adverse reactions.

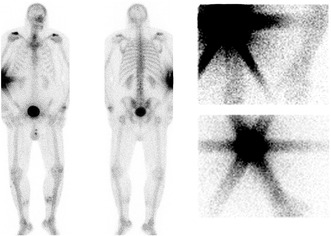

**Abbreviations**
[99mTc]Tc‐HDP: [99mTc]Tc‐hydroxy‐diphosphonate
IU: International Unit

## 1 INTRODUCTION

[99mTc]Tc‐hydroxy‐diphosphonate ([99mTc]Tc‐HDP) is a widely used radiopharmaceutical for skeletal imaging. It binds to calcium ions as hydroxyapatite, leading to specific uptake in osteoblastic bones, making it highly effective for detecting bone metastases in cancer and diagnosing various bone‐related conditions.^1^ Compared with therapeutic radiopharmaceuticals, extravasation of imaging agents like [99mTc]Tc‐HDP typically results in minimal harm due to limited radiation exposure. Consequently, few guidelines address the management of extravasations involving imaging radiopharmaceuticals.^2^

Despite the generally low risk, extravasations can still cause adverse effects, including local tissue damage and discomfort.^3^ This short report details an instance of [99mTc]Tc‐HDP extravasation and explores the use of hyaluronidase to mitigate the associated risks. The report aimed to highlight the importance of recognizing and appropriately managing such events to minimize patient harm and improve outcomes in nuclear medicine practice.

## 2 CASE PRESENTATION

**FIGURE 1:**
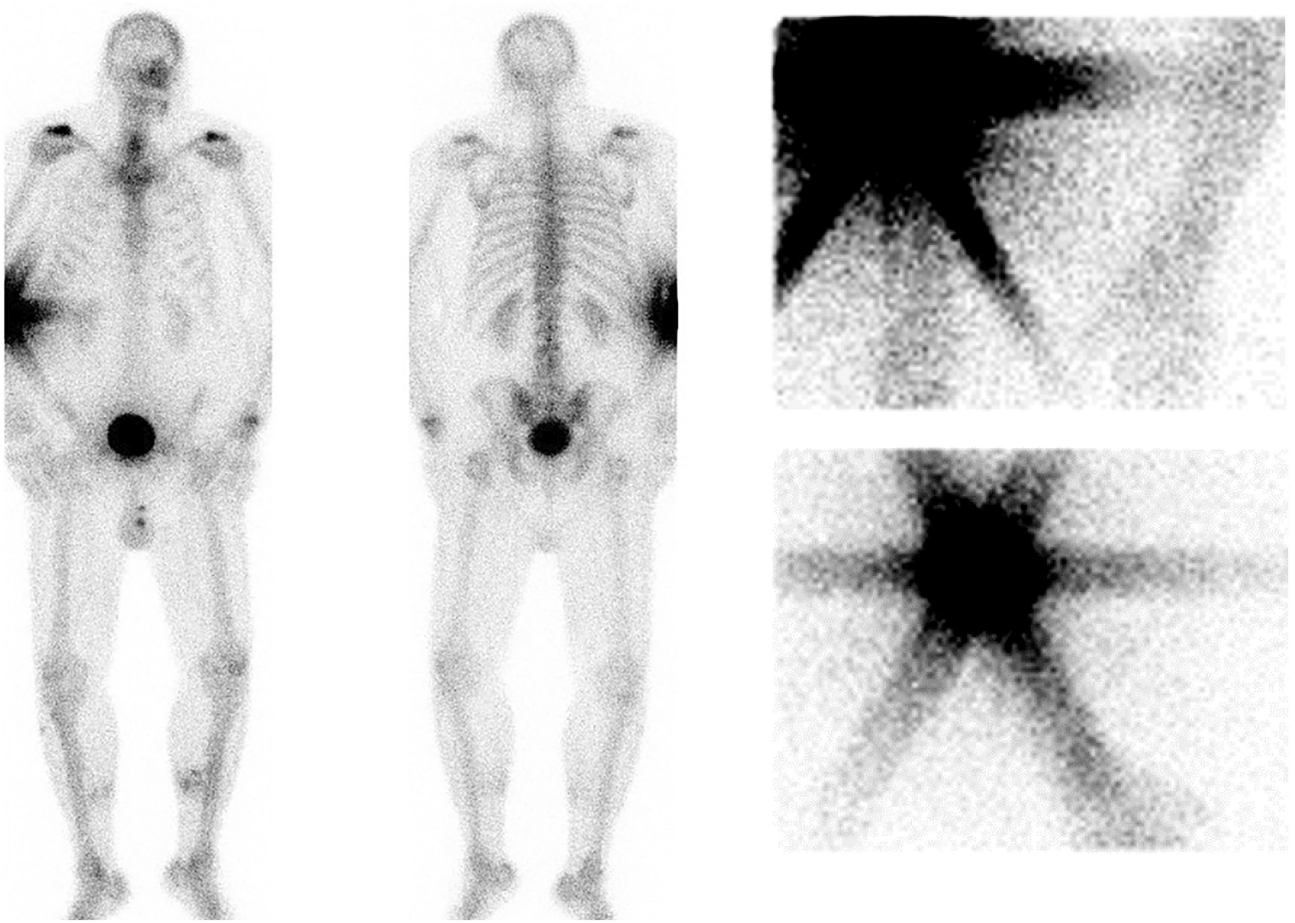
High activity at the site of extravasation in the right elbow is illustrated on anterior and posterior (AP) total‐body planar scintigraphy images 4 h post‐extravasation (left); and anterior images of the (AP) planar imaging of the right elbow injection site used for consequent dosimetry calculation, at 30 min (top right) and 4 h (bottom right).

A 50‐year‐old male patient presented with pain and dysfunction 1 year after a left‐sided distal radius fracture. In order to better diagnose and treat the patient, 871 MBq [99mTc]Tc‐HDP (volume 6 mL, body weight 130 kg) was administered to perform three‐phase bone scintigraphy.^1^ After intravenous injection, dynamic imaging showed no visual activity at the wrist. This prompted the technician to suspect an extravasation. This extravasation was subsequently investigated further using planar imaging of the injection site (Figure 1).

Standard saline flushing and aspiration of the extravasation site could not be performed due to the butterfly syringe having been dislodged during administration. Given the high activity concentration and colloid (i.e., poorly spreading) nature of [99mTc]Tc‐HDP, we ordered elevation of our patients' arm (above the heart level) with warm compresses at the extravasation site and consulted the plastic surgeon on‐call. The plastic surgeon injected hyaluronidase (150 IU) to facilitate in spreading the radiopharmaceutical by enzymatic degradation of local interstitial matrix components. Additional planar acquisitions were performed 4 h post‐injection for dosimetry purposes. These were considered suboptimal for diagnostic purposes, leading to a second three‐phase bone scintigraphy being performed at a later date. Initial swelling and redness at the extravasation site persisted after 1 week and fully resolved at 3‐month follow‐up.

## 3 DISCUSSION

Extravasation of Tc‐99m labeled radiopharmaceuticals is generally considered to cause no serious adverse events and require no specific intervention.^2^ Even so, the absorbed radiation dose is not always negligible and other characteristics such as the pH of the radiopharmaceutical can also cause adverse reactions.^4^ To assess potential risks, dosimetry was used in accordance with the Medical Internal Radiation Dose (MIRD) Pamphlet No. 21^5^; absorbed dose to a target tissue (i.e., skin) is given as: $\dot{D}\left(r_{T},t\right)=\sum_{r_{s}}\tilde{A}\left(r_{S},t\right)\times S\left(r_{T}\leftarrow r_{S},t\right)$. Here, $\tilde{A}\left(r_{S},t\right)$ is the time‐integrated activity of the injected dose at the injection site, and $S\left(r_{T}\leftarrow r_{S},t\right)$ is the mean absorbed dose rate per unit activity from source to target region. Assuming no biological clearance from the injection site takes place, the time integrated activity can be calculated by dividing the administered activity by the decay constant of the isotope: $\frac{871\mathrm{MBq}}{\ln\left(2\right)/6}\approx 7540\;\mathrm{MBq}\;h$.

If no additional measures had been taken to facilitate spreading of the injected 6 mL of [99mTc]Tc‐HDP (compresses and hyaluronidase), the maximum absorbed dose to the skin can be estimated using the spheres module in IDAC‐dose to be 13 Gy (S(6 mL skin) = 1.749 mGy/MBq h).^6^

With the additional measures taken to facilitate spreading of [99mTc]Tc‐HDP, including injection of 150 IU hyaluronidase to enzymatically degrade the local interstitial matrix; we estimated that the effective half‐life of the radiopharmaceutical at the extravasation site was reduced to 1.5 h, and consequently $\tilde{A}\left(r_{S},t\right)$ = 1885MBqh.^7^ Assuming no change in the distribution volume, this would result in a total activity dose of 3.3 Gy. When assuming that hyaluronidase increased the volume to 10 mL, the estimated absorbed dose is reduced to 2.0 Gy. These calculated absorbed dosages are in line with dose ranges found in previous studies using Monte Carlo simulations.^7^, ^8^

In retrospect, we are unsure whether the local skin redness and swelling that persisted for the first weeks after extravasation as observed in our patient was related to the absorbed dose (2.0–3.3 Gy) or chemically induced (acidic low pH).^4^, ^9^, ^10^ Nonetheless, we propose that more serious local reactions may be prevented using measures including elevation, warm compresses, and safe 150 IU hyaluronidase injection after extravasation of high‐activity, low‐volume, colloidal [99mTc]Tc‐based radiopharmaceuticals such as [99mTc]Tc‐HDP.

## AUTHOR CONTRIBUTIONS

Kevin R. Doornhof is responsible for the content. Dr. Quido de Lussanet de la Sablonière is the radiologist in charge of the patient's treatment at the time of the extravasation and provided crucial information about the actions taken; Dr. Stijn L. W. Koolen is the hospital pharmacist overseeing the use of radiopharmaceuticals at the Erasmus MC. Dr. Mark W. Konijnenberg performed the dosimetry calculations related to the extravasation. All authors contributed their insights and provided feedback during the writing of this report.

## CONFLICT OF INTEREST STATEMENT

The authors declare that there are no conflicts of interest to disclose.

## ETHICS STATEMENT

The Medical Research Ethics Committee (MREC) of the Erasmus Medical Centre was contacted during preparation of this report. The committee determined that ethics review and approval was not required for its publication.

## CONSENT

Written informed consent was obtained from the patient for the publication of this report and the accompanying images.

## Data Availability

The data that support the findings of this study are available from the corresponding author upon reasonable request.
